# Crystal-field mediated electronic transitions of EuS up to 35 GPa

**DOI:** 10.1038/s41598-022-05321-9

**Published:** 2022-01-24

**Authors:** Virginia Monteseguro, Jose A. Barreda-Argüeso, Javier Ruiz-Fuertes, Angelika D. Rosa, Holger L. Meyerheim, Tetsuo Irifune, Fernando Rodriguez

**Affiliations:** 1 DCITIMAC, MALTA Consolider Team, Facultad de Ciencias, University of Cantabria, 39005 Santander, Spain; 2 ESRF, The European Synchrotron, 71 Avenue des Martyrs, 38000 Grenoble, France; 3 Max-Planck-Institut für Mikrostrukturphysik, Weinberg 2, 06120 Halle, Germany; 4 Ehime University, 2-5 Bunkyo-cho, Matsuyama, 790-8577 Japan; 5 Earth-Life Science Institute, Tokyo Institute of Technology, Tokyo, 152-8500 Japan

**Keywords:** Materials science, Condensed-matter physics, Electronic properties and materials

## Abstract

An advanced experimental and theoretical model to explain the correlation between the electronic and local structure of Eu$^{2+}$ in two different environments within a same compound, EuS, is presented. Eu*X* monochalcogenides (*X*: O, S, Se, Te) exhibit anomalies in all their properties around 14 GPa with a semiconductor to metal transition. Although it is known that these changes are related to the $4f^7 5d^0$ $\rightarrow$ $4f^6 5d^1$ electronic transition, no consistent model of the pressure-induced modifications of the electronic structure currently exists. We show, by optical and x-ray absorption spectroscopy, and by *ab initio* calculations up to 35 GPa, that the pressure evolution of the crystal field plays a major role in triggering the observed electronic transitions from semiconductor to the half-metal and finally to the metallic state.

## Introduction

Eu$^{2+}$ has always attracted scientists of different fields in Chemistry, Physics and Materials Science and Technology due to its unique electronic structure characterized by its $4f^7 5d^0$ ground and $4f^6 5d^1$ excited state. Eu$^{2+}$ is one of the most investigated activator in highly efficient phosphors^1–3^. The high oscillator strength of the $4f^7 5d^0$ $\rightarrow$ $4f^6 5d^1$ electric-dipole transition makes it easy to excite it under blue-UV light^4^. Furthermore, the outer 5*d* electrons strongly interact with the surrounding ions (ligands) allowing the tunability of the photoluminescence wavelength from red to blue just by selecting the appropriate host site structure^1,5^. Eu*X* monochalcogenides(*X*: O, S, Se, Te) offer an excellent opportunity to study the pressure dependence of the correlation between the electronic and the geometric structures in Eu$^{2+}$, especially in view of the simple cubic structures and regular local environments. Eu*X* crystallizes in the NaCl-type structure (B1, space group $Fm{\bar{3}}m$), and undergoes a phase transition to the high-pressure (hp) CsCl-type structure (B2, space group $Pm{\bar{3}}m$) under compression. Thus, Eu$^{2+}$ experiences an increase of the coordination from octahedral (Eu$X_6$) to hexahedral (Eu$X_8$). The transition pressure ($P_C$) is higher as the ionic radius of *X* decreases. The EuO, EuS, EuSe and EuTe series undergoes the structural phase transition at 50, 21.5, 18 and 10.5 GPa, respectively.

For the group of Eu*X* monochalcogenides intriguing (pressure-induced) magnetic, electronic and optical modifications have been observed during the last decades. Their magnetic order is sensitive to subtle structural ligand-induced changes in the atomic Eu-*X*-Eu superexchange chain, which gives rise to competing ferro- and antiferromagnetic coupling with neighbouring ions. While EuO and EuS are ferromagnetic with Curie temperatures ($T_C$) of 67 K and 16.2 K, respectively^6–11^, EuSe ($T_C$ = 16.2 K) is ferrimagnetic^7^ and EuTe anti-ferromagnetic ($T_N$ = 16.2 K)^8^. It was suggested that the different magnetic ordering in Eu*X* is related to the elongation of the Eu–*X*–Eu atomic chain^7–9,11^, which is sensitive to pressure-induced modification. For instance, antiferromagnetic EuTe can be transformed into ferromagnetic by applying an external pressure above 8 GPa^8^. Interestingly, the high-pressure dependence of $T_C$ in Eu*X* exhibits strong positive trends, especially above 14 GPa^12^.

The most studied compound of the series, EuO, undergoes a sudden resistivity drop^13^ and an unusual reflectivity increase^14^ under external pressure of 14 GPa. These anomalies have been tentatively explained by a change of the oxidation state of Eu$^{2+}$ under lattice compression, either to Eu$^{3+}$^15^ or to a mixed valence state^10,16^, via $f-d$ hybridization and by the overlap of the *d*-states with the anion (*X*) *np* orbitals. However, we are missing a well-founded explanation to those anomalies at $\sim$14 GPa. A recent theoretical work in EuO^17^ reported two electronic transitions at 16 and 33 GPa. The former is attributed to an insulator–metal transition, while the latter to a metal–metal transition, although only the second transition is discussed in detail. Notably, this latter is predicted to be due to a different pressure evolution of the population of 5*d*($t_{2g}$) and 5*d*($e_g$) orbitals^17^. However, the pressure-induced modification of the crystal-field splitting (CFS) and its effect on the EuO electronic structure taking place at 14 GPa have not been explained so far.

Despite all these efforts to disentangle the correlation between pressure and electronic properties in Eu*X* compounds, a thoroughly experimental study of the electronic structure covering a wide pressure range well beyond the B1–B2 phase transition is not available so far. The study of the electronic structure modified by external pressure is challenging. It requires to carry out optical and X-ray spectroscopy experiments by using a diamond anvil cell^18^ with micro-focus beam system at a pressure beyond 21 GPa. This difficulty increases in optical absorption experiments due to the intense background scattering in EuS powders^19^.

In this work we present a comprehensive and detailed experimental analysis of the pressure dependence of the electronic structure of EuS up to 35 GPa by optical absorption and XANES spectroscopy. The experimental results are supported by ab initio calculations within the density functional theory (DFT) formalism. We show that the crystal-field (CF) strength at the Eu$^{2+}$ site plays a crucial role in the modification of the electronic structure in EuS. Likewise, this conclusion can be extended to the whole Eu*X* series.

The width of the optical gap (EuS $4f \rightarrow 5d$) was studied by optical absorption experiments, while the CFS and the Eu oxidation state was probed by XANES spectroscopy within the stability regime of the B1 and B2 phases. The experimental results were confirmed by calculations of the band structure and the partial density of states (p-DOS) as a function of pressure. The calculated pressure evolution of the band structure elucidates the relative energetic position of the valence orbitals (4*f* and 5*d*) of Eu$^{2+}$ in octahedral coordination.

## Results and discussion

**Figure 1:**
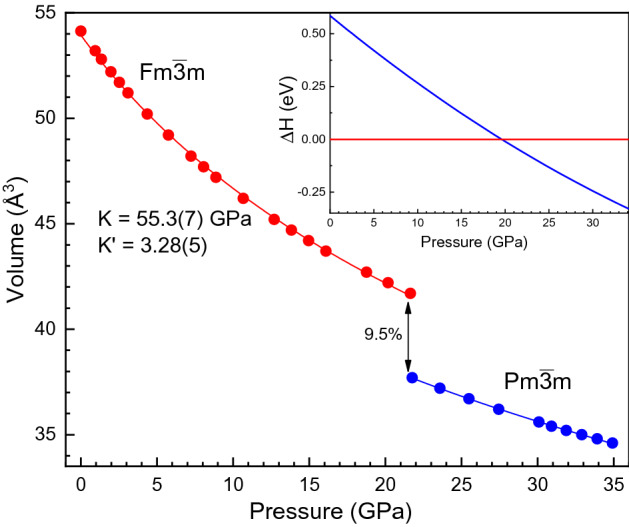
Volume-pressure curve up to 35 GPa showing the volume collapse of 9.5% at the B1–B2 phase transition at 21.5 GPa. The enthalpy-pressure curves are represented in the inset.

X-ray diffraction (XRD) experiments indicates that at ambient pressure EuS crystallizes in the cubic $Fm{\bar{3}}m$ phase, NaCl-type, B1, with lattice parameter of 5.97(2) and a bulk modulus, $K_0$, of 61.5(5) GPa^20^. It is worth noting that our calculations provide a precise description of the evolution of the EuS crystal structure under pressure, as can seen in the Supplementary Fig. S1.1, yielding a lattice parameter of *a* = 6.01(2) Å(*V* = 54.27(6) Å$^3$/Eu) and a $K_0$ = 55.3(7) GPa with $K^{'}$ = 3.28(5) (Fig. 1). Moreover, our calculations predict a volume collapse, around 9.5%, at the same experimental $B1 \rightarrow B2$ transition pressure, $P_C$ = 21.5 GPa. Our calculated structural phase transition is also confirmed by enthalpy-pressure curves shown in the inset of Fig. 1.

**Figure 2:**
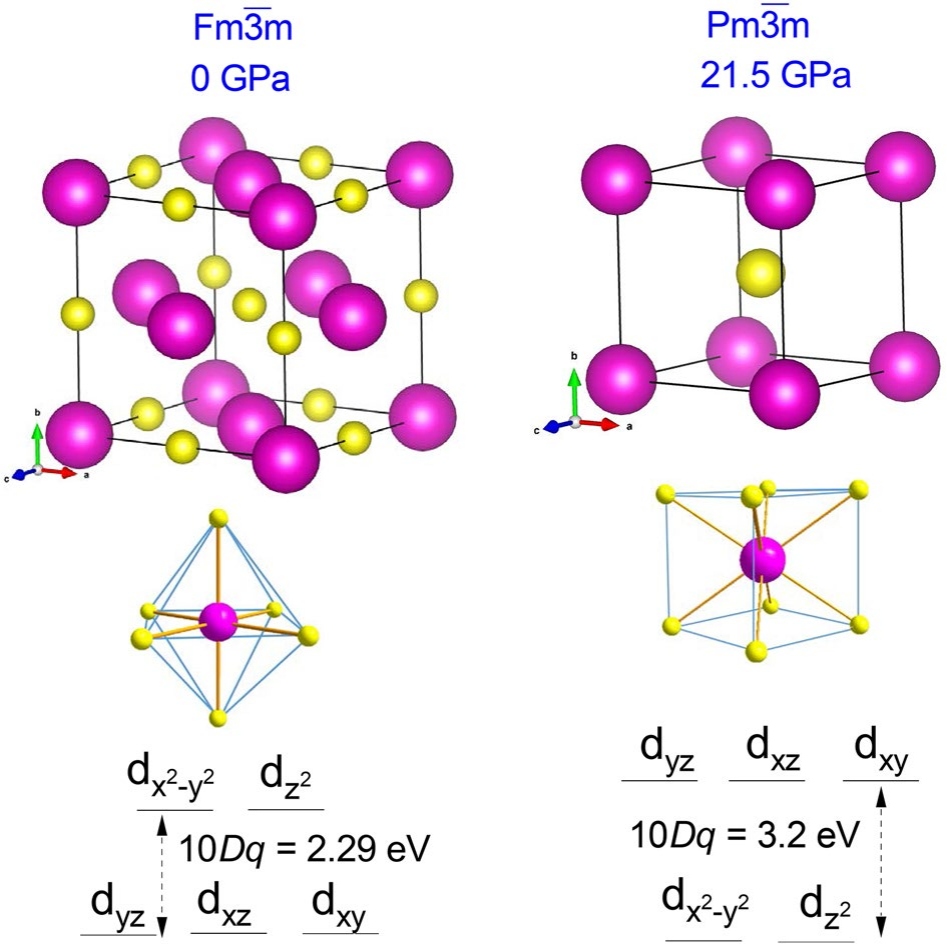
Unit cell and Eu$^{2+}$–S coordination in the B1 (left) and B2 phase (right). Pink and yellow balls represent Eu$^{2+}$ and S$^{2-}$ ions, respectively. The respective energetic positions of the $5e_g$ and the $5t_{2g}$ orbitals are schematically shown next to the structure models.

A coordination change from octahedral, EuS$_6$, to hexahedral, EuS$_8$, takes place in the B1-B2 transition (Fig. 2). It is worth noting that the modification of the coordination geometry correlates with the CFS between $e_g$ and $t_{2g}$ orbitals. While in the NaCl-type structure (B1 phase), the $t_{2g}$ orbitals ($d_{xy}$, $d_{xz}$, and $d_{yz}$) are lower in energy than the $e_g$ orbitals ($d_{x^2-y^2}, d_{z^2}$), the situation is reversed in the CsCl-type structure (hp B2 phase). This is schematically shown in Fig. 2. Here, the CFS is also indicated as 10*Dq* in line with usual notation.

**Figure 3:**
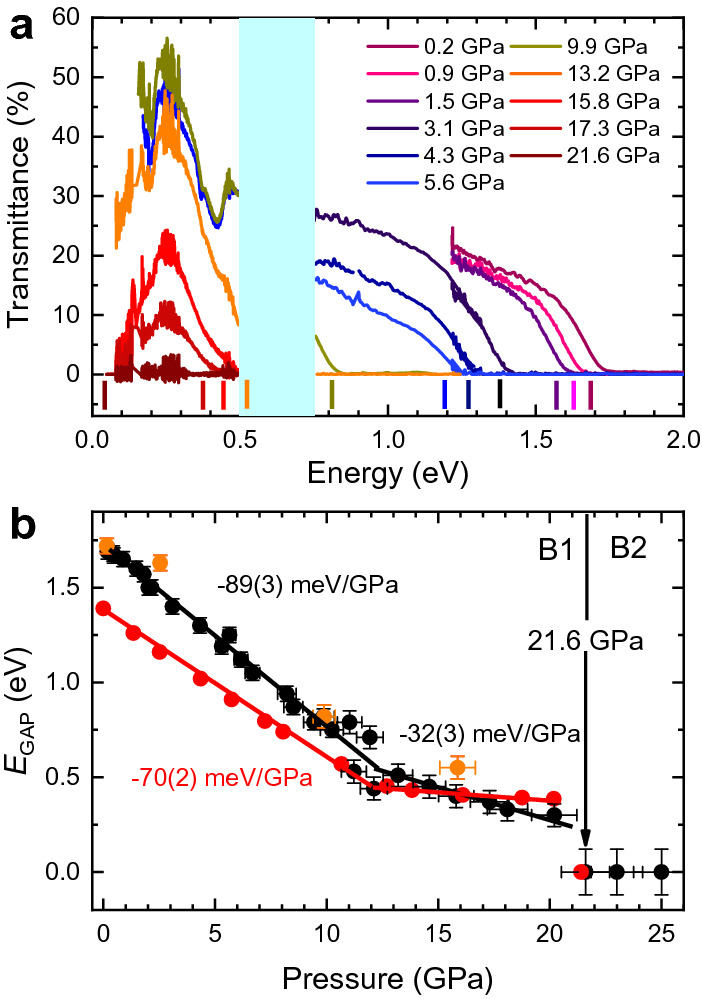
(**a**) Pressure dependence of the optical transmittance in EuS in the 0–25 GPa range at room temperature. The spectral range spans 0.05–0.5 eV and 0.74–3 eV. (**b**) Experimental up-stroke/downstroke (black/orange symbols) and calculated (red symbols) represent the direct band gap width at the *X*-point. Lines are guides to the eye.

Figure 3a shows the pressure dependence of the transmittance in the visible-IR spectral range between 0 and 25 GPa in EuS in order to better illustrate the complete metallization above 21.5 GPa. The direct optical gap, $E_{GAP}$, characterized by the onset of the $4f^7 5d^0 \rightarrow 4f^6 5d^1$ electric-dipole transition, has been determined from the optical spectra by means of Tauc plots^21^. For further information about the determination of $E_{GAP}$ see the Supplementary Information (Supplementary Fig. S2.1a,b). Its value of 1.72 eV at ambient pressure matches with those reported elsewhere^22,23^. Remarkably, its pressure dependence unveils three different regimes as shown in Fig. 3b, where the experimental and calculated values of $E_{GAP}$ are represented with black and red symbols, respectively.

From 0 to 13.5 GPa the band gap decreases at a rate of − 89(3) meV GPa$^{-1}$. At 13.5 GPa we find $E_{GAP}$ = 0.5 eV, above this pressure the slope ($\partial E_{GAP}/ \partial P$) being reduced to − 32(3) meV GPa$^{-1}$ up to 21.5 GPa. Finally, $E_{GAP}$ becomes zero above 21.5 GPa along with the B1–B2 phase transition. These three different regimes are also observed in the optical transmittance under pressure, *T(P)*, represented in Supplementary Fig. S2.2.

These three different regimes reflect how the charge transfer between the Eu 4*f* and 5*d* is affected by applying pressure. A very similar pressure dependence of the optical and electronic properties has been also observed in the other europium monochalcogenides. For instance, the electrical resistivity in EuO exhibits a continuous drop with increasing pressure in agreement with theoretical predictions, while a sudden reduction is observed at about 14 GPa^13^. Similarly, the optical reflectivity increases with pressure showing also a sudden increase at 14.4 GPa. This rise of reflectivity was initially explained in terms of a progressive pressure-induced Eu$^{2+}$ $\rightarrow$ Eu$^{3+}$ valence change^14,15^. However, later investigations by Mössbauer spectroscopy^16^ and XANES measurements^10^ proposed a different charge-transfer mechanism based on an admixture of Eu$^{2+}$ and Eu$^{3+}$ ground state. The resistivity drop at 14 GPa was related to a hybridized-valence band mixed-valence semiconductor phase^13^. Nevertheless, this interpretation lacked experimental evidence from a pressure-dependent optical absorption study of the direct optical band gap in EuO.

**Figure 4:**
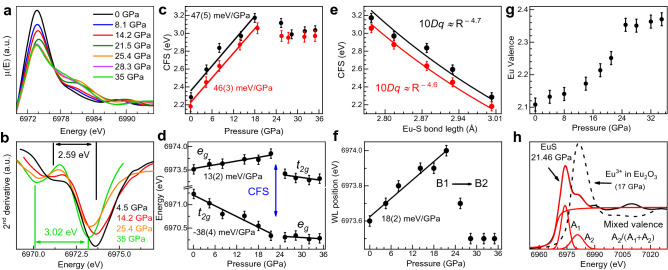
(**a**) XANES spectra between 0 to 35 GPa. (**b**) Pressure evolution of $\partial ^2 \mu (E)/ \partial E^2$ showing the minima whose energetic separation corresponds to the CFS as indicated (**c**) experimental (black dots) and theoretical (red dots) CFS versus pressure. (**d**) Experimentally derived energetic position of the Eu $5t_{2g}$ and $5e_g$ orbitals versus pressure. (**e**) Observed (black dots) and theoretical (red dots) CFS versus Eu-S bond length in the B1 phase. (**f**) Observed pressure evolution of the WL position. (**g**) Experimental Eu valence state under lattice contraction. (**h**) Spectral weight approach to obtain the oxidation state. The XANES spectra were fit by arctan + two Gaussians. The areas $A_1$ and $A_2$ of the Gaussians fitted to the absorption spectrum are related to the relative fraction of Eu$^{2+}$ and Eu$^{3+}$.

The variation of the XANES spectra of EuS collected in the vicinity of the Eu $L_3$-edge (6975 eV) with pressure is shown in Fig. 4a. All XANES spectra show an intense WL associated with the electric-dipole transition from the $2p_{3/2}$ to the $5d_{3/2,5/2}$ levels, which probes the local density of unoccupied 5*d* states split by the CF, i.e. the CFS.

As already outlined by Laguna-Marco et al.^24^, the CFS of Eu$^{2+}$ 5*d* orbitals can be derived quantitatively throughout exhaustive analysis of the white line (WL) of the Eu$^{2+}$ $L_3$-edge which corresponds to the $2p_{3/2} \rightarrow 5d_{3/2,5/2}$ transition. The second derivative of the WL unveils the two components, the energetic separation corresponds to the CFS between the $5t_{2g}$ and $5e_g$ levels^24^ (Fig. 4c). At ambient conditions the experimental (black symbols) CFS is equal to 2.29 eV, in agreement to the CFS values obtained by optical absorption^25^ (Fig. 4c). Under pressure, it increases at a rate of 46(5) meV GPa$^{-1}$ up to the $B1 \rightarrow B2$ phase transition pressure at 21.5 GPa, reaching a value of 3.2 eV. Above 21.5 GPa the CFS remains nearly constant within the pressure range of the B2 phase: 10*Dq* $\approx$ 3 eV. Its evolution within the B1 phase unravels a different pressure dependence of the energy of the two WL components associated with the $5t_{2g}$ and $5e_g$ orbitals such as outlined in Fig. 4d. The $5t_{2g}$ levels decrease in energy at a rate of − 38(4) meV GPa$^{-1}$ while the $5e_g$ levels increase at 13(2) meV GPa$^{-1}$. In terms of the Eu-S bond distance (*R*) dependence, 10*Dq* scales as $R^{-n}$ with measured and calculated *n* exponents in B1 of 4.7(5) and 4.6(3), respectively (Fig. 4e). Both are close to *n* = 5, typical value of the variation of CFS of under pressure in octahedral coordinated *d* ions. The CFS reduction across the B1–B2 phase transition is caused by the lengthening of the Eu-S bond (inset of Fig. 1) although it is partially compensated by the increase of CFS by the 6 to 8 coordination change (Fig. 2). The pressure-induced Eu-S length contraction in the B1 phase also yields a shift of the WL centroid towards higher energies at a rate of 18(2) meV GPa$^{-1}$ (Fig. 4f)).

The Eu valence has been determined (Fig. 4g) through the spectral weights of Eu$^{2+}$ ($4f^7$) and Eu$^{3+}$ ($4f^6$) following the procedure outlined in Fig. 4h)^26^. The energetic position of the Gaussian *A*2 has been compared with the WL position of Eu$_2$O$_3$ (Eu$^{3+}$) at a pressure of 17 GPa close to $P_C$. The variation of the Eu valence from 2.10 at 0 GPa to 2.35 at 21.5 GPa bares a mixed-valence state, but in no way, the process involves a full valence change to Eu$^{3+}$ neither in the B1 nor in the B2 phase. Therefore, this result coincides with previous interpretations mentioned above based on XANES studies performed on EuO and EuSe^10^ and Mössbauer spectroscopy measurements in EuO^16^.

**Figure 5:**
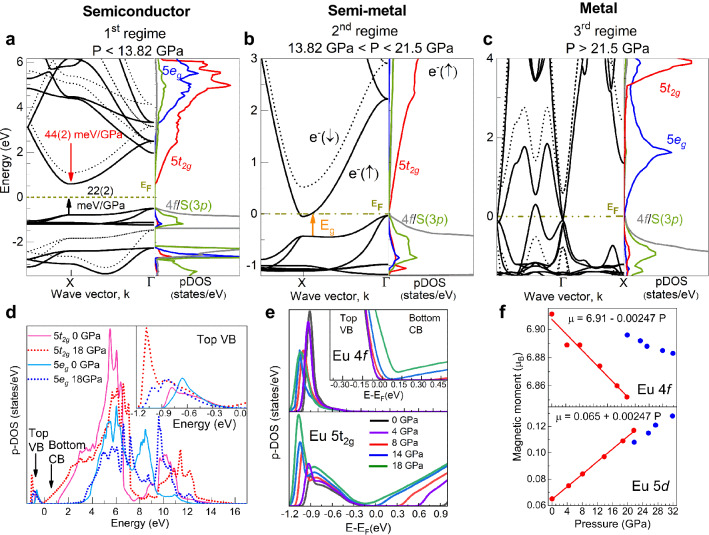
(**a**) Band structure and p-DOS of EuS, around $E_F$, at 0 GPa in the semiconductor phase, (**b**) at 13.8 GPa in the half-metal phase and (**c**) at 21.5 GPa in the metal phase. Solid and dashed lines correspond to spin-up and spin down states, respectively. The onset of the half-metal phase (**b**) is characterized by the closure of the indirect gap $\Gamma \rightarrow X$, the transition to the metallic phase (**c**) by the gap closure at $\Gamma$. Note the inversion of the energetic position of the $t_{2g}$ and the $e_g$ band during the half-metal to metal phase transition which is also evident in (**d**) showing the p-DOS of the Eu $5t_{2g}$ and $5e_g$ orbitals in the range between − 1.2 and 16 eV. (**e**) p-DOS of the Eu 4*f* and $5t_{2g}$ states in the pressure range between 0 and 18 GPa. The inset shows a detail of the Eu 4*f* DOS. At 18 GPa the Eu $5t_{2g}$ level has a finite DOS at $E_F$ indicating the onset of metallization. (**f**) Orbital-resolved magnetic moment of 4*f* and 5*d* levels versus pressure.

Figure 5 shows the calculated band structure and the p-DOS of EuS at 0, 13.8 and 21.5 GPa (plots a, b and c, respectively), corresponding to the three observed $E_{GAP}$(*P*) regimes. The section of band structures and p-DOS shown correspond to that around the Fermi energy in order to observe clearly the phenomena occurring during the metalization processes. The whole band structures and entire p-DOS are presented in Supplementary Fig. S3.1 and S3.2, respectively. In EuS, the top of the valence band (VB) is formed by hybridized Eu 4*f* and S 3*p* spin-up orbitals represented by solid lines (dashed lines correspond to spin-down orbitals). The bottom of the conduction band (CB) consists mainly of Eu $5t_{2g}$ and $5e_g$ spin-up orbitals. With increasing pressure, the energetic position of the CB minimum at the *X*-point (the Eu $5t_{2g}$ subband) decreases at a rate of − 44(2) meV GPa$^{-1}$, similar to the experimental decrease of the $5t_{2g}$ band (Fig. 4d). Simultaneously, the bands of the top of the VB (Eu 4*f*–S 3*p* subband) increase in energy at a rate of 22(2) meV GPa$^{-1}$, equivalent to the increase of the WL position due to the shortening of the Eu-S bond length with pressure. Overall, this leads to a pressure-induced gradient of the theoretical energy gap, $E_{GAP}$, of − 70(2) meV GPa$^{-1}$, being consistent with the experimental result (− 89(3) meV GPa$^{-1}$) within the first regime (red symbols in Fig. 3a).

**Figure 6:**
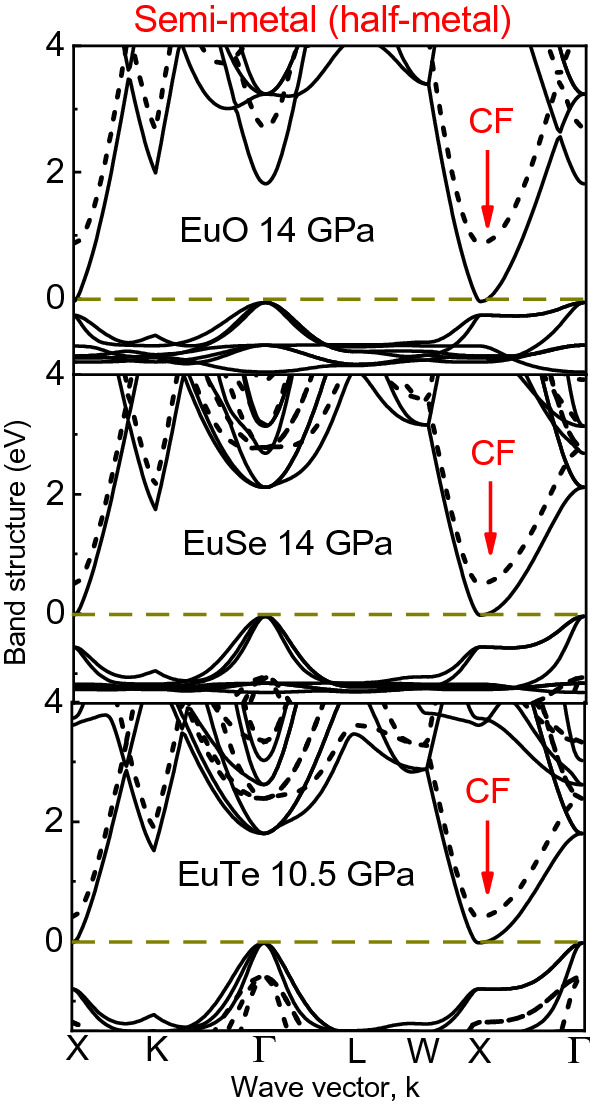
Band structure of EuO (up), EuSe (middle) and EuTe (down) at 14 GPa and 10.5 GPa, respectively, at the semiconductor to half-metal transition.

At 13.8 GPa the bottom of the CB at the *X*-point intercepts the Fermi level ($E_F$) thereby leading to the closure of the indirect band gap ($\Gamma \rightarrow X$). This indirect band gap closure corresponds to the electronic transition from semiconductor to half-metal. The latter is characterized by a half-metallic state, involving the overlap of the $5t_{2g}$ spin-up orbitals (CB minimum) with the partially hybridized Eu 4*f*–S 3*p* spin-up orbitals (top of VB) below $E_F$. The band structure of the spin-down orbitals (dashed lines in Fig. 5a–c) still corresponds to that of a semiconductor^27^. The halfmetallicity is responsible for the reduced experimental pressure gradient of $E_{GAP}$(P) in the 13.8–21.5 GPa range (Fig. 3b). In the second regime of $E_{GAP}$, the $4f \rightarrow 5d$ transition does not occur from the top of VB to the bottom of CB. Instead, the electron transfer during excitation is from the top of VB to the first empty state above $E_F$ as indicated by the orange arrow ($E_{GAP}$) in Fig. 5b. Further pressure increase closes $E_{GAP}$ at the *X*-point due to a half-metal to metal transition. The same metallization process at similar pressure points has been observed in other sulphur chalcogenides such as MoS$_2$ by resistivity measurements and also confirmed by ab initio calculations^28^. It must be noted that recently a similar electronic transition at 33 GPa was observed in EuO and interpreted as due to a different pressure evolution of the $5t_{2g}$ and $5e_g$ orbital population^17^. However, such a model cannot be employed in EuS as the second electronic transition (half-metal to metal) occurs along with the structural B1 $\rightarrow$ B2 phase transition at 21.5 GPa (Figs. 1 and 2), where also the energetic position of the $5t_{2g}$ and $5e_g$ orbitals is inverted (Figs. 2 and 4d). As a result, the $5e_g$ orbitals are now located at the bottom of the CB (p-DOS of Fig. 5c).

Within the B1 phase, the different pressure evolution of the $5t_{2g}$ and $5e_g$ orbitals derived through XANES (Fig. 4d) is also confirmed by the calculations carried out in the -1.6–16 eV energy range as it is shown in the p-DOS of the $5t_{2g}$ and the $5e_g$ orbitals (red and blue lines in Fig. 5d).The solid and dashed lines in this plot correspond to 0 and 18 GPa, respectively. The unoccupied p-DOS of the $5t_{2g}$ orbitals above $E_F$ becomes broader under pressure as it approaches to the bottom of CB, while the unoccupied pDOS of the $5e_g$ orbitals barely undergo significant changes (Fig. 5d). In the top of VB (inset of Fig. 5d), the number of occupied states of $5t_{2g}$ orbitals rises with pressure while this number for $5e_g$ orbitals remains almost constant. The theoretical CFS derived from p-DOS matches suitably with the experimental one (Fig. 4c).

The progressive electron delocalization of the 4*f* states with pressure is evidenced through their p-DOS (Fig. 5e, up). The top of VB shifts towards higher energy whereas the lower VB decreases in energy yielding a broadening of the Eu 4*f* states. It must be noted that as the delocalization of the 4*f* states evolves, there is an increase in occupancy of $5t_{2g}$ states (Fig. 5e, down). Interestingly, as pressure increases, the orbital-resolved magnetic moment of 4*f* and 5*d* levels decreases and increases, respectively, at the same rate of ± 2.47(6) $\times$ 10$^{-3}$ μB GPa$^{-1}$ (Fig. 5f). The overlap between 4*f* and 5*d* orbitals has been also observed in EuO and EuSe^11^. Therefore, our theoretical results unveils a charge transfer mechanism from 4*f* to 5*d* orbitals, specifically to the $5t_{2g}$ levels. Within our model, the theoretical pressure evolution of the $4f^7 5d^0 \rightarrow 4f^6 5d^1$ electric-dipole transition described above (Fig. 3b) perfectly reproduces the experimental one. In other words, the pressure dependence of the calculated $E_{GAP}$ agrees with the experimental dependence shown in Fig. 3b. Indeed, the charge transfer between 4*f* and 5*d*($t_{2g}$) orbitals is likely relevant to explain why the $5t_{2g}$ levels rapidly drop in energy compared to the $5e_g$ orbitals, which exhibit an opposite trend, both effects leading to the important increase of the CFS in EuS under pressure (Fig. 5a,b).

We have also calculated the electronic band structures of EuO and the other Eu*X* monochalcogenides, which are shown in Fig. 6. The orbital description of the bands is the same in the compound series. The top of the VB consists of hybridized Eu 4*f*–*Xnp* orbitals and the bottom of the CB of Eu 5*d* states. Moreover, the semiconductor to half-metal transition originated by the rise of the CFS is a common feature taking place around 14 GPa. One exception to this rule is EuTe whose first electronic transition occurs at 10.5 GPa and is concomitant with the B1 $\rightarrow$ B2 phase transition. Interestedly, our predicted band gap closure at 10.5 GPa in EuTe may explain the change in the trend of its resistivity above 10 GPa recently observed by Li et al.^29^. These results confirm that the electronic properties of EuS and the Eu*X* series closely resemble each other with regard to the band structure as well as to their pressure-induced modifications. Therefore, the resistivity drop^13^ and the reflectivity rise^14^ at 14 GPa in EuO can be likely due to the delocalization of the 4*f* states and the consequent increase of occupied $5t_{2g}$ states due to the first semiconductor-halfmetal electronic transition.

## Methods

### Optical absorption spectroscopy

The experiments were carried out in an Almax–Boehler diamond anvil chamber (DAC). Optical absorption spectroscopy experiments under hp conditions were carried out using a prototype fiber-optics microscope equipped with two reflecting objectives of $25\times$ magnification mounted on two independent x–y–z translational stages for the microfocus beam, and the collector objective. A third independent x–y–z translational stage was used for the DAC holder^30^. Spectra in the UV-VIS and near infrared were recorded with Ocean Optics USB 2000 and NIRQUEST 512 monochromators using Si- and InGaAs-CCD detectors, respectively, both covering a spectral range from 300 to 1700 nm (4–0.74 eV). For IR transmission measurements under pressure, we used a Thermo Nicolet FTIR Microscope, equipped with suitable reflecting objectives of 20 × magnification for focusing and collecting light. Spectral resolution was 0.1 cm$^{-1}$. Transmission spectra *T*($1/\lambda$) = $I(1/\lambda )/I_0(1/\lambda )$, were obtained by measuring the transmitted intensity passing through the sample (*I*) and through the pressure transmitting medium ($I_0$). For this purpose, we used parallelepipedal samples of about $70\times 70$ $\mu m^2$ in size to allow the rectangular light spot with dimension of $50\times 20$ $\upmu m^2$ to fully pass though the sample. For optical absorption, an Almax–Boehler-type DAC equipped with ultralow fluorescence diamond anvils was used. The pressure cell was loaded with a 20 $\upmu m$ thick pellet of EuS powder, ruby sphere as a pressure sensor and KBr as a pressure transmitting medium.

### X-ray absorption spectroscopy.

X-ray absorption spectroscopy (XAS) experiments were carried out at the BM23 beamline^31^ of the European Synchrotron Radiation Facility (ESRF) equipped with a double crystal Si (111) monochromator and KB mirrors with a Pt coating to focus the x-ray beam to a spot of $5\times 5$ μm$^2$ in size. The x-ray incidence angle on to the mirrors was fixed to 6.5 mrad to eliminate higher harmonics. X-ray absorption spectra were collected in transmission mode in the vicinity of the Eu $L_3$- (6975 eV) and the $L_2$-edge (7617 eV) as a function of pressure up to 35 GPa using a membrane-type pressure cell (DAC) equipped with 300 μm culet nanopolycrystalline diamond anvils^32^. As pressure transmitting medium methanol–ethanol–water (16–4–1) was used. Pressure was determined through the luminescence of a ruby placed inside the cell chamber. The XANES profiles were fitted by the sum of an arctangent and a Lorentz function^33^ in order to obtain the integrated intensities and the energy position of WL associated to the $L_3$ and the $L_2$ edges as a function of pressure. The modification of the pressure-induced Eu valence state was derived by fitting the relative changes of the spectral weights ($A_1$ and $A_2$) of the Eu$^{2+}$ ($4f^7$) and Eu$^{3+}$ ($4f^6$) contribution of the XANES. The valence state modification is then estimated by using the formula $A_2/(A_1+A_2)$ where $A_1$ and $A_2$ are the areas of the Gaussian peaks in the curve fitting of the WL and the first oscillation, respectively^26^.

### Ab initio calculations

They were performed within the density functional theory (DFT) formalism^34^. We used the VASP package to perform calculations with the pseudopotential method and the projector augmented wave scheme^35,36^. For Eu, $5s^2 5p^6 4f^7 5d^{(0)} 6s^2$ electrons were treated as valence electrons, for S all six valence electrons ($3s^2 3p^4$) were taken into account. Highly converged results were achieved by extending the set of plane waves up to a kinetic energy cutoff of 450 eV. The local spin-density approximation with a strong intratomic interaction (GGA + U) in the Dudarev’s approach was used in order to correctly describe the strongly correlated Eu 4*f* electrons^37^. The onsite Coulomb interaction U was set to 7 eV and the exchange parameter *J* to 0.6 eV, both yielding reliable results of the lattice parameters and magnetic moments as compared to the experimental ones. We have used the PBE description^38^ within the GGA aproximation for the exchange-correlation energy. At each selected volume (pressure), the structure was fully relaxed to its equilibrium configuration through the calculation of the forces on atoms and of the stress tensor. A dense $9 \times 9 \times 9$ *k*-point mesh was used to perform integrations within the Brillouin zone. In the relaxed configurations, the forces on the atoms are less than 0.006 eV Å$^{-1}$ and the deviation of the stress tensor from a diagonal hydrostatic form is less than 0.1 GPa. The electronic total and partial density of states were calculated by doubling the *k*-points grid used previously in the relaxation.

## Conclusions

Two changes were unraveled in the pressure evolution of E$_{GAP}$ by optical absorption spectroscopy up to 25 GPa: first, a change of the pressure gradient of the $E_{GAP}$ around 13.8 GPa, and secondly, a complete metallization at 21.5 GPa. According to our band structure and p-DOS calculations, the first anomaly is attributed to a closure of the indirect band gap ($\Gamma \rightarrow X$) corresponding to the electronic transition from semiconductor to half-metal. A full metallization of EuS takes place at 21.5 GPa along with the B1 $\rightarrow$ B2 structural phase transition. This second electronic transition from half-metal to metal is triggered by the closure of the direct optical band gap at the *X*-point.

We demonstrated by XANES spectroscopy that the increase of the CFS between $5d(t_{2g})$ and $5d(e_g)$ orbitals is the driving force of the two observed electronic transitions. The CFS increases in the B1 phase as a result of the different pressure dependence of the $5t_{2g}$ and $5e_g$ orbital energy. The energy of the $5t_{2g}$ orbitals decreases rapidly with pressure (the Eu–S shortening), while the $5e_g$ levels show an opposite trend. The band structure and p-DOS calculations confirm the opposite pressure gradient of the orbitals.The fast drop of the $5t_{2g}$ levels is likely due to the pressure-induced charge transfer from the Eu 4*f* to the 5*d* ($5t_{2g}$) levels during the metallization of EuS.

Finally, we showed that the mixed-valence state Eu$^{2.35+}$ in the B2 phase of EuS is in agreement with findings in EuO and EuSe^10,16^. Band structure calculations in EuO, EuSe and EuTe confirm the close resemblance in electronic properties of the Eu*X* series. Therefore, our experimental and theoretical description of the electronic structure and its evolution with pressure in terms of short and long range order structural changes around Eu$^{2+}$ in EuS, can be adequately transferred to other Eu monochalcogenides.

## Supplementary Information

Supplementary Information.
