# Supplementary material for: Crystal-field mediated electronic transitions of EuS up to 35 GPa
Source: Sci Rep. 2022 Jan 24;12:1217. doi: 10.1038/s41598-022-05321-9 (PMC8786971; doi:10.1038/s41598-022-05321-9)
Supplement: Supplementary file 1 — Supplementary Information. [file 41598_2022_5321_MOESM1_ESM.pdf]

Supplementary information for:

Crystal-field mediated electronic transitions of EuS up to 35 GPa

Virginia Monteseguro*,†, José Antonio Barreda-Argüeso†, Javier Ruiz-Fuertes†, Angelika Rosa‡, Holger L. Meyerheim¶, Tetsuo Irifune§,ǁ and Fernando Rodríguez†

†*MALTA Consolider Team, DCITIMAC, Facultad de Ciencias, Universidad de Cantabria, Avda. de Los Castros s/n, 39005, Santander, Spain.*

‡*ESRF, The European Synchrotron, 71 Avenue des Martyrs, 38000 Grenoble, France*

¶*Max-Planck-Institut für Mikrostrukturphysik, Weinberg 2, D-06120 Halle, Germany.*

§*Ehime University, 2–5 Bunkyo-cho, Matsuyama, 790-8577, Japan.*

ǁ*Earth-Life Science Institute, Tokyo, Institute of Technology, Tokyo, 152-8500, Japan*

1. Pressure dependence of the unit-cell volume

The pressure evolution of the calculated volume (in red) obtained by our *ab initio* calculations is compared in Fig. S1.1 with previous experimental data. Interestingly, our theoretical structural volume data under compression are in very good agreement with the experimental results.

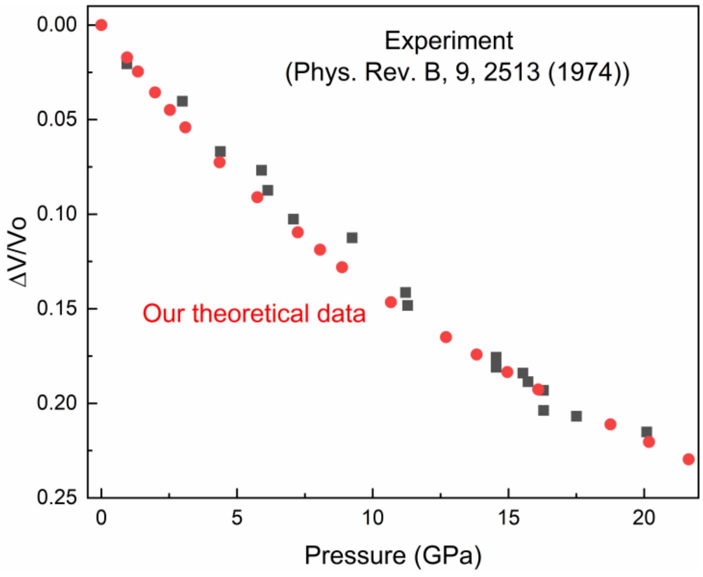

Figure S1.1. Theoretical (our work, in red) and experimental (Ref. 1, in black) pressure-volume data in the B1 phase of EuS.

2. Experimental energy gap determination

The determination of the direct gap as a function of pressure was achieved through Tauc plots from the absorption spectra. The absorption spectra were determined from the transmittance spectra by the equation: $A(E) = -\log [\mathrm{T}(E)]$, with E being the photon energy of light: $E = \mathrm{h}c/\lambda$, and $A(E)$ the absorbance, or optical density, at E. Absorbance can be transformed to absorption coefficient units by the formula: $\mathrm{a}(E) = 2.3\, A(E)/l$, where l is the light path that is $l = 20$ mm $= 2.10^{-3}$ cm at ambient pressure, and must be corrected by the factor $(V/V_0)^{1/3}$ using the equation of state of EuS at every pressure P. The measured $A_{\mathrm{m}}(E)$ contains two main contributions, the first one is $2\log[1\text{-R}(E)]$, and the second one is $\mathrm{a}(E)\, l\, /\, 2.3$, given that the ratio of intensities is in first approximation: $I/I_0 = (1\text{-}R)^2 \exp(-\mathrm{a}(E)\ l)$. Therefore, the experimentally obtained absorbance $A_{\mathrm{m}}(E) = -\log [I/I_0]$ in a semiconductor or an insulator consists of an absorption background, $A_0(E)$, containing the first term due to the reflectivity and other scattering processes, plus a steep absorption due to the direct optical band gap absorption, $\mathrm{a}(E)\, l\, /\, 2.3$. Therefore, the experimentally obtained absorbance can be written as $A_{\mathrm{m}}(E) = -\log [I/I_0] = \mathrm{A}_0(E) + A(E)$ from which the absorption coefficient, $\mathrm{a}(E)$, is proportional to $A(E)$ and is derived from $A_{\mathrm{m}}(E)$ by $A(E) = A_{\mathrm{m}}(E) - A_0(E)$. This equation is used to extract the direct optical band gap from the absorption spectra.

As it is illustrated in Fig. S2.1 the direct gap energy, E_{GAP}, was determined from Tauc plots –plot of either $A(E)^2$ or $\mathrm{a}(E)^2$ vs. E [2, 3]– by fitting the band gap threshold points ($E > E_{\mathrm{GAP}}$) to the linear equation: $\mathrm{C}(E\text{-}E_{\mathrm{GAP}})$, with C and E_{GAP} being the fit parameters. This procedure provides E_{GAP} accuracies in EuS better than 0.05 eV in the whole pressure range. The error was determined from the standard deviation of the corresponding fit parameter.

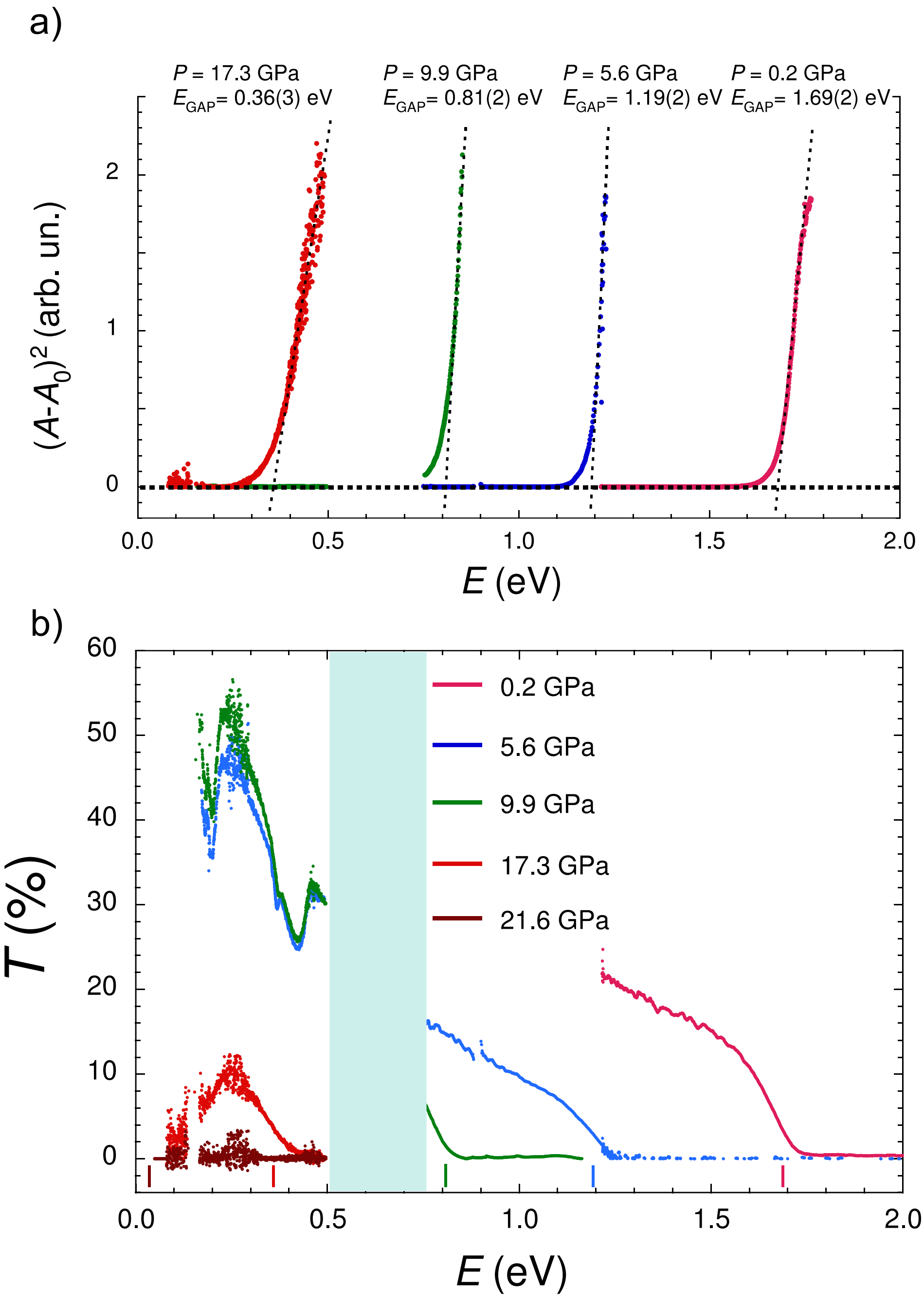

Figure S2.1. a) Tauc plots for EuS at selected pressures. Note that absorption background $A_0(E)$ is taken from the below gap low-energy points by a least-square linear fitting. b) Corresponding transmittance spectra with bars indicating the E_{GAP} values derived in a).

Figure S2.2 shows the variation of optical transmittance in the IR region. In EuS the plot of the transmittance at 0.1 and 0.3 eV vs. pressure unravels the gap closure and the two different metallization regimes found in the analysis of experimental $E_{GAP}(P)$ values and by DFT methods. Although the errors in the transmittance analysis are significantly bigger than in $E_{GAP}(P)$, both variations confirm the metallization processes described in the manuscript.

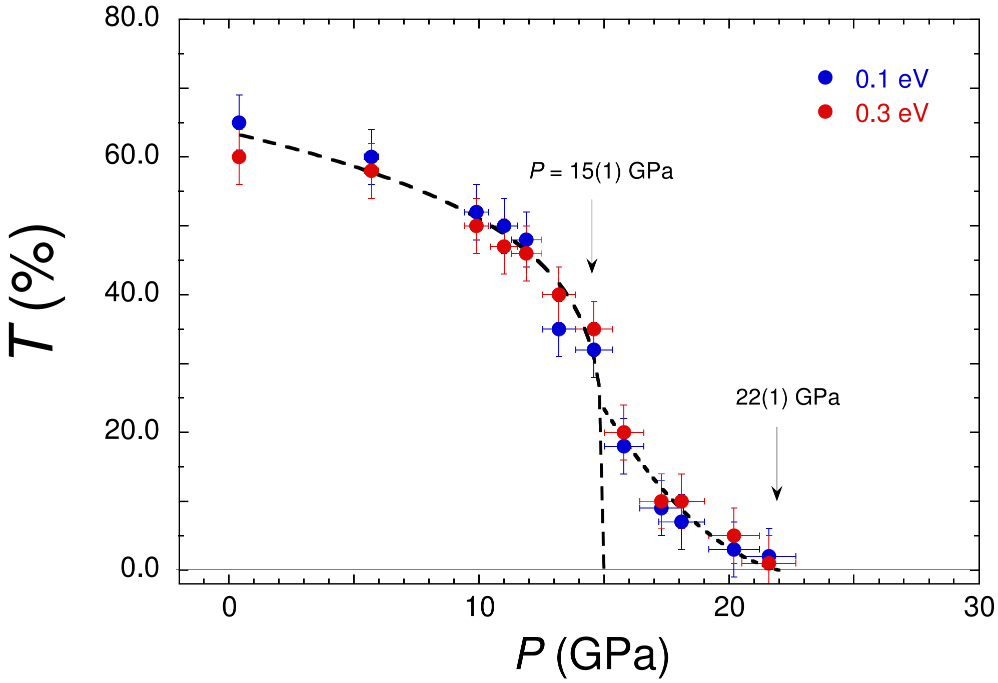

Figure S2.2. Variation of the optical transmittance at 0.1 and 0.3 eV with pressure in EuS. Note the three different pressure regimes of the variation $T(P)$. The dotted lines are phenomenological curves described as a function of (P_{M1}-P), and (P_{M2}-P), respectively. The values of P_{M1} and P_{M2} are included in the figure.

3. Band structure and projected electronic density of states

The whole band structure at 0 GPa, 14 GPa, and 21.5 GPa are shown in Figure S3.1. The three electronic states of EuS can be observed, the semiconducting state at 0 GPa (Fig. S3.1a), the semimetallic state at 14 GPa (Fig. S3.1b) in the low pressure B1 phase, and the metallic state at 21.5 GPa in the high-pressure B2 phase (Fig. S3.1c). The entire

projected electronic density of states are shown in Figure S3.2 at 0 GPa (a), 14 GPa (b) and 21.5 GPa (c).

a)

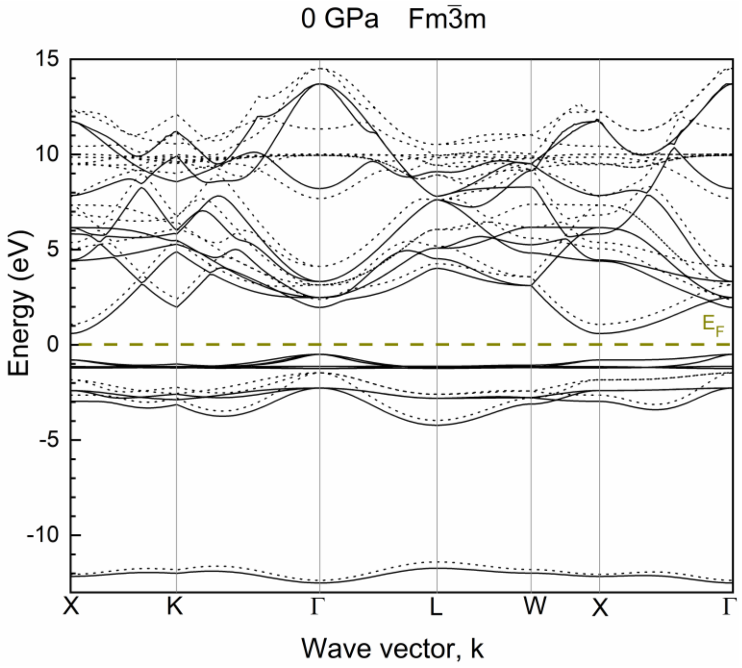

b)

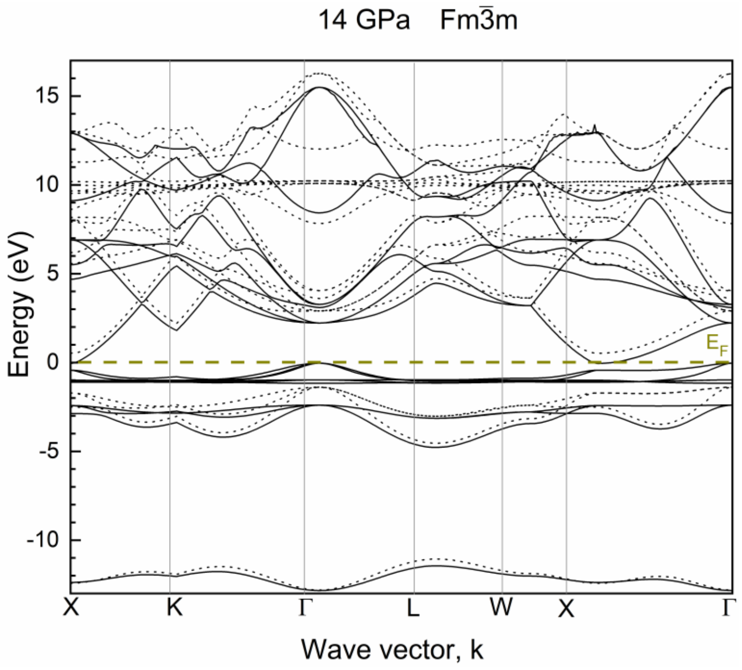

c)

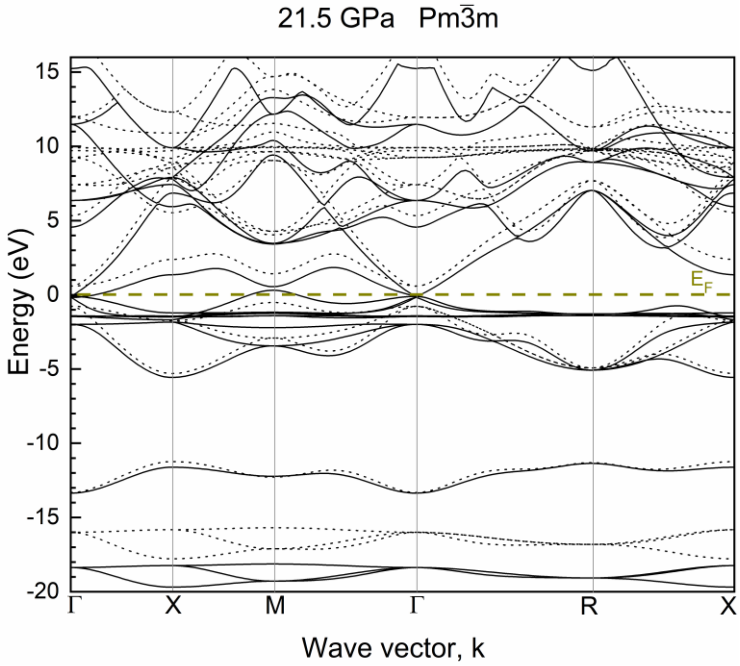

Figure S3.1. Band structure at 0 GPa (a), 14 GPa (b), and 21.5 GPa (c). (a) and (b) correspond to the B1 phase and (c) corresponds to the B2 phase. The solid (dot) lines represent the spin-up (spin-down) bands.

a)

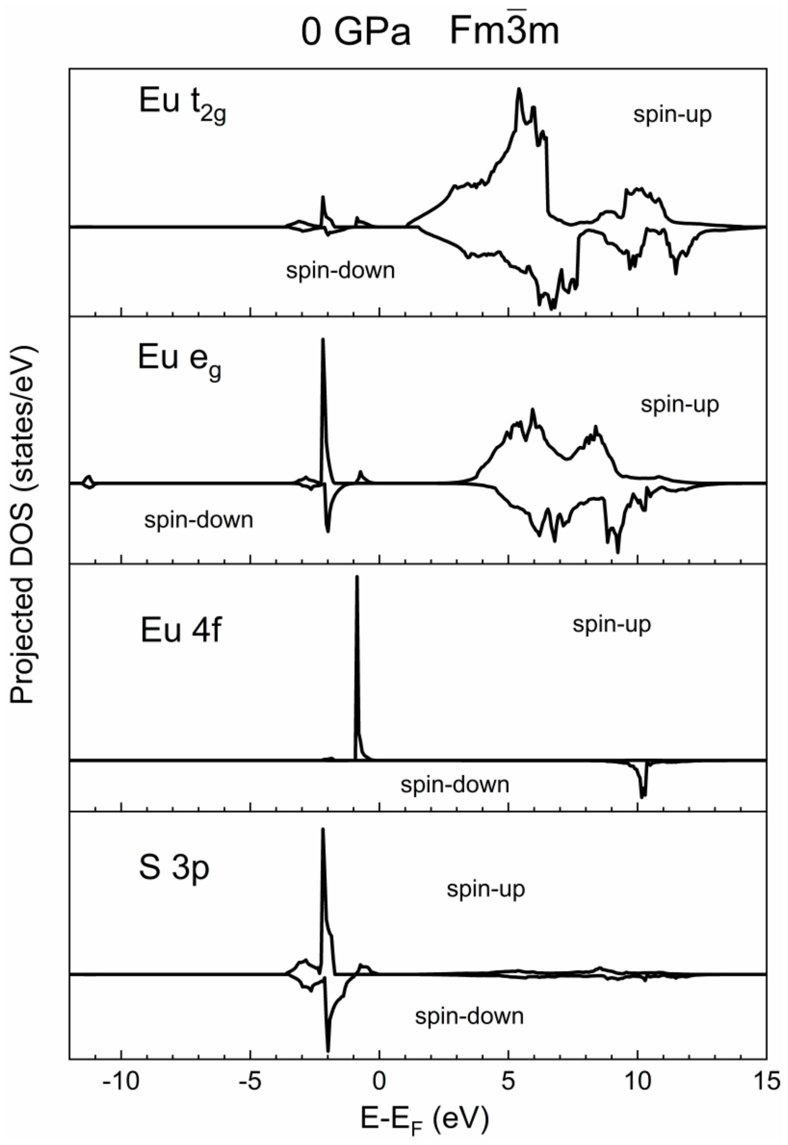
0 GPa Fm$\bar{3}$m
Eu t2g
spin-up
spin-down
Eu eg
spin-up
spin-down
Eu 4f
spin-up
spin-down
S 3p
spin-up
spin-down
Projected DOS (states/eV)
-10
-5
0
5
10
15
E-EF (eV)

b)

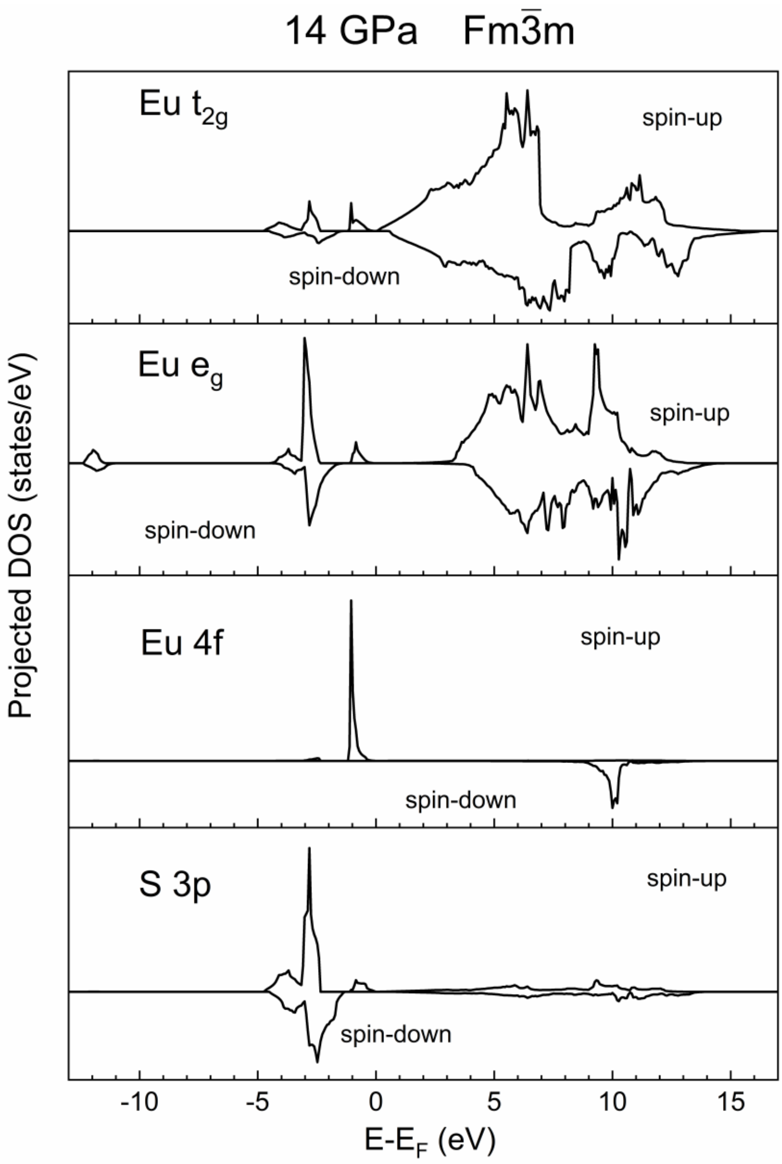
14 GPa Fm$\bar{3}$m
Eu t2g
spin-up
spin-down
Eu eg
spin-up
spin-down
Eu 4f
spin-up
spin-down
S 3p
spin-up
spin-down
Projected DOS (states/eV)
-10
-5
0
5
10
15
E-EF (eV)

c)

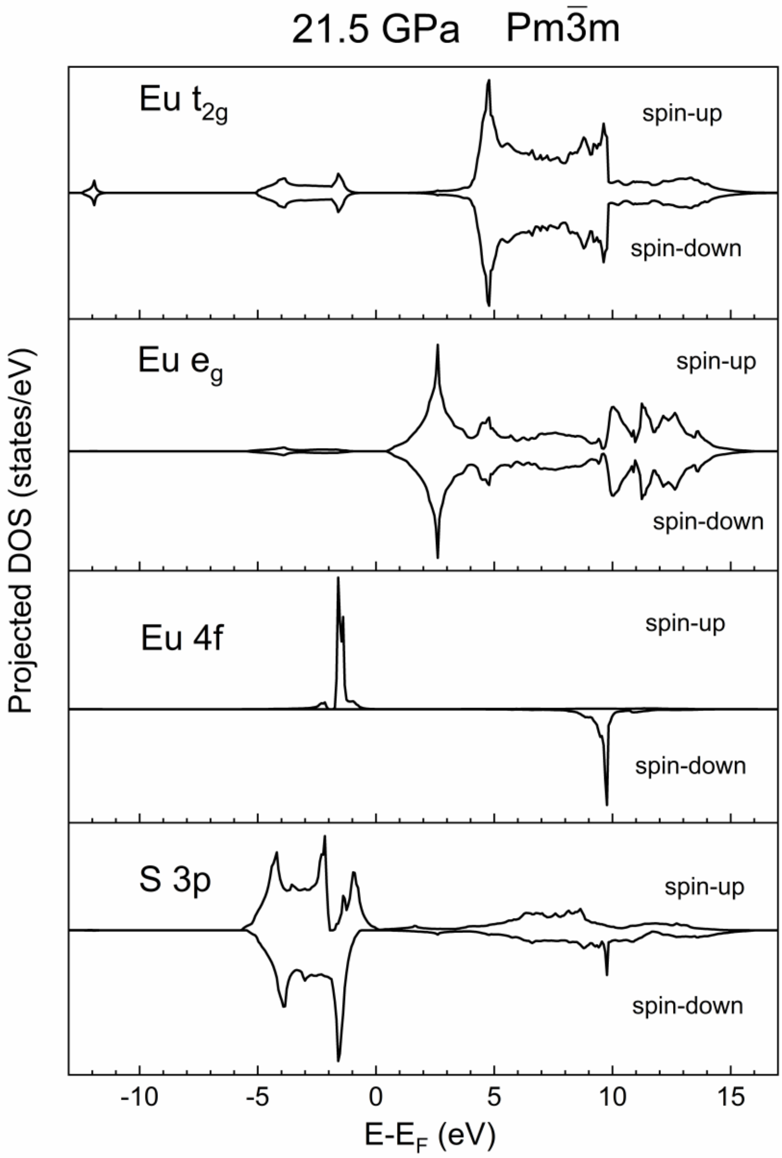

Figure S3.2. Projected electronic density of states (DOS) at 0 GPa (a), 14 GPa (b), and 21.5 GPa (c). The two former correspond to the B1 phase and the latter correspond to the B2 phase.

References

[1] A. Jarayaman, A. K. Singh, A. Chatterjee, and S. Usha Devi, *Phys. Rev. B*, 1974, **9**, 2513.

[2] J. Tauc, Optical properties and electronic structure of amorphous Ge and Si, *Mat. Res. Bull.*, 1968, **3**, 37.

[3] M. Fox, *Optical Properties of Solids*, Oxford University Press, New York, 2001.
